# The Relationship between Change-of-Direction Performance Indicators and Inter-Limb Asymmetries in Elite Youth Female Basketball Players

**DOI:** 10.5114/jhk/202104

**Published:** 2025-04-30

**Authors:** Jordi Arboix-Alió, Bernat Buscà, Mariona Peralta-Geis, Alicia M. Montalvo, Azahara Fort-Vanmeerhaeghe

**Affiliations:** 1 Department of Sports Sciences, Ramon Llull University, FPCEE Blanquerna, Barcelona, Spain.; 2 School of Health Sciences, Ramon Llull University, FCS Blanquerna, Barcelona, Spain.; 3 FC Barcelona, Sport Performance Area, Barcelona, Spain.; 4 College of Health Solutions, Arizona State University, Phoenix, AZ, USA.; 5 Segle XXI Female Basketball Team, Catalan Federation of Basketball, Esplugues de Llobregat, Spain.

**Keywords:** imbalances, ground contact time, sprint, jumping, symmetry

## Abstract

The purposes of this study were to characterize the magnitude and the direction of inter-limb asymmetries in elite youth female basketball players and to evaluate any associations with indicators of change-of-direction (COD) performance. Eighteen elite youth female basketball players (17.79 ± 0.67 years; 71.10 ± 7.43 kg; 1.82 ± 0.07 m; 23.01 ± 1.69 kg∙(m^2^)^−1^) participated in a battery of tests, including 70° and 180° COD, single-leg countermovement jump (SLCMJ), and single-leg drop jump (SLDJ) tests. Inter-limb asymmetry indices (ASIs) were calculated, and correlations with COD performance indicators were examined. The results indicated significant differences between dominant and non-dominant limbs in all tasks, with ASI values ranging from 3.02% to 27.8%. Directionality of asymmetry varied across tests. Correlation analysis revealed that greater asymmetry was associated with slower COD performance (ρ range = −0.67 to 0.57). Moreover, asymmetry in some tasks was related to lower ground reaction forces (GRFs) (ρ range = −0.60 to −0.42) and higher contact times during COD 180° (ρ = 0.45). The results of this study support the need to address inter-limb asymmetries in basketball players. Coaches and practitioners should consider targeted interventions to minimize asymmetry and enhance COD skills. A comprehensive battery of fitness assessments is recommended to provide a holistic understanding of inter-limb asymmetries.

## Introduction

Women’s team sports have seen a surge in popularity. Basketball is not an exception, and, lately, the number of studies with female samples in the sport has grown considerably (Power et al., 2022; Reina et al., 2020). Basketball is a physically demanding sport that challenges athletes' agility and change-of-direction (COD) abilities, which are inherent to the game (Banda et al., 2019; Castagna et al., 2007). Effective performance in COD tasks, such as cutting, pivoting, and quick direction changes, are crucial for success on the court. These movements require athletes to generate high forces, decelerate and accelerate rapidly, and maintain stability and balance (Reina et al., 2020; Vázquez-Guerrero et al., 2018). Thus, strength and conditioning practitioners are constantly working to create new techniques to enhance players’ skill and performance (Loturco et al., 2019).

Inter-limb asymmetries are differences in performance between an individual's dominant and non-dominant limbs and may influence performance (Bishop et al., 2021a). Recently, several studies have reported the frequency of inter-limb asymmetries in various fitness tests among healthy athletes (Bishop et al., 2018; Fox et al., 2023). While there is some evidence of inverse associations between asymmetries and jump height (Bell et al., 2014), acceleration (Bishop et al., 2021b), sprint performance (Arboix-Alió et al., 2020), COD performance (Madruga-Parera et al., 2021), and flywheel resistance skills (Fort-Vanmeerhaeghe et al., 2022a) in team sport athletes, studies aggregating results are inconclusive (Bishop et al., 2018).

Female athletes tend to exhibit higher asymmetries relative to male athletes, which can result in performance differences, injury risk, and long-term athletic development (Fort-Vanmeerhaeghe et al., 2022b). These differences are believed to be influenced by factors such as hormonal fluctuations, differences in body composition, and differences in neuromuscular activation patterns (Fort-Vanmeerhaeghe et al., 2021a). Moreover, it is also important to explore specific COD variables that may be affected by inter-limb asymmetries, such as vector forces and ground contact times (GCTs) (Arboix-Alió et al., 2024; Maloney et al., 2015).

Although prior studies have investigated asymmetry within COD speed tasks, the emergence of cutting-edge technology and instruments, such as force plates, has ushered in fresh avenues for exploration (Thomas et al., 2020). These force plates signify a pivotal advancement in the field, offering an unprecedented level of precision and detail. They provide the means to gather exceptionally precise measurements of ground reaction forces (GRFs) during COD movements (Dos’Santos et al., 2020; Maloney and Fletcher, 2021), and to quantify forces exerted in each limb during a variety of physical tests, including COD tasks (Maloney et al., 2017). Additionally, force plates enable the meticulous examination of other variables, such as vectors, mechanical efficiency or the timing of contact forces, thereby shedding light on the temporal aspects of asymmetry in COD performance. Such detailed information by limb, particularly during a COD task, is useful for identifying factors that may contribute to asymmetry (Bishop et al., 2021a).

This study had two primary objectives: 1) to assess the size and the direction of asymmetries in elite youth female basketball players through jumping and COD tests, and 2) to quantify the association between asymmetries and indicators of COD performance in the same population. We hypothesized that the size and the direction of inter-limb asymmetries would differ between limbs, and that higher inter-limb asymmetry would correlate with lower physical performance outcomes in COD tests.

## Methods

We used a cross-sectional design to investigate the associations between inter-limb asymmetries and COD performance indicators in a group of elite youth basketball players. We conducted the fitness testing battery at the start of the post-season over two consecutive days. Inter-limb asymmetries were calculated for single-leg countermovement jump (SLCMJ), single-leg drop jump (SLDJ), and 70º and 180º COD tests, which allowed for comprehensive examination of unilateral vertical jumping and COD limb differences.

### Participants

We recruited 18 elite youth female basketball players using convenience sampling for this study (age: 17.79 ± 0.67 years, body mass: 71.10 ± 7.43 kg, body height: 1.82 ± 0.07 m, body mass index: 23.01 ± 1.69 kg∙(m^2^)^−1^, sports experience: 6.42 ± 1.41 years). We performed a power analysis in advance using G*Power (Version 3.1, University of Düsseldorf, Germany) which indicated that we needed at least 16 participants to achieve a statistical power of 0.8 with an alpha threshold of 0.05 for type I errors. Since our study involved 18 participants, the statistical power was approximately 0.75. At the time of the study, all participants were engaged in a four-year talent development program. Their weekly schedule typically consisted of 7–9 training sessions, along with a weekend game. This study was approved by the Ramon Llull University Ethics Committee, Barcelona, Spain (approval code: 1718007D; approval date: 25 February 2021) and conformed to the recommendations of the Declaration of Helsinki (revised in Fortaleza, Brazil, 2013).

### Design and Procedures

We familiarized participants with all tests and procedures one week prior to data collection. On testing days, participants completed a standardized warm-up routine. The routine started with a 5-min general cardiovascular warm-up (RPE 5–6). Next, participants completed 6 min of multidirectional movements, followed by 4 min of dynamic stretching exercises, and concluded with a 3-min phase of high-intensity movement that progressed in difficulty. Participants were given three practice trials to learn the tests, and performed at effort levels of 70%, 85%, and 100%, respectively, of their maximum capacity. A 2-min rest period was provided between the last practice trial and the start of the first test.

### COD Tests

**Figure 1:**
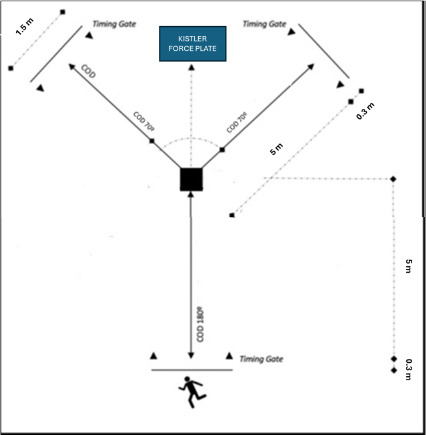
Schematic representation of the change direction tests.

For the COD measurements, we instructed participants to complete a COD task consisting of a combination of a 5-m run and a 70° or a 180º turn to the finish line situated 5 m from the COD point (Figure 1). The COD times in seconds were recorded with photoelectric cells (Witty, Microgate, Bolzano, Italy). Players were instructed to start with their dominant foot in front and 0.5 behind the first gate (Nimphius et al., 2016). Gates were spaced 1.5 m apart and rose 1.3 m off the ground. Each participant completed two trials, then rested for 3 min between each attempt. The fastest trial in each direction was used in the analyses..

To collect ground reaction force (GRF) data during the change-of-direction (COD) test, we instructed athletes to cut so that the outside foot made full contact within the boundaries of the force plate. We discarded trials where that foot landed outside the force plate's area. GRFs in all three dimensions (x-axis, y-axis, and z-axis), along with ground contact time (GCT), were recorded using a force plate (Kistler 9260AA, Winterthur, Switzerland) paired with a data acquisition system (Kistler 5695b, Winterthur, Switzerland). We used the MARS software (Kistler, Winterthur, Switzerland) for data acquisition and storage at a sampling rate of 1000 Hz and for calculating all dependent variables. We calibrated the system according to the instructions provided by the MARS software.

### Single Leg Countermovement Jump

The SLCMJ test was conducted using a contact mat (Chronojump Boscosystem, Barcelona, Spain) (Bosco et al., 1983). We recorded flight time with Chronojump software (version 1.9.0) to calculate vertical jump height. For the SLCMJ test, participants were instructed to balance on one leg with their hands on their hips. We then instructed them to perform a countermovement to a comfortable depth, followed by a rapid extension of the stance leg to achieve maximum vertical height during the jump. Throughout the flight phase, participants kept the test leg fully extended until landing on the contact mat. We asked participants not to swing the opposite leg prior to the jump. Participants were given clear instructions to exert maximal effort and “jump as high as possible” for each trial. To ensure consistency, participants kept their hands on their hips throughout the movement. A trial was considered successful if this criterion was met. Each athlete completed three trials on each leg, and the maximum jump height achieved on each side was used for further data analysis.

### Single Leg Drop Jump

The SLDJ test was carried out using a contact mat system (Chronojump Boscosystem, Barcelona, Spain). To start, we positioned participants on an 18-cm box (Bishop et al., 2022a). Clear instructions were given to athletes to step off the box, maintain hands on hips, and land on the same limb. Throughout the jump, we directed them to “minimize ground contact time and maximize jump height” (Bishop et al., 2022a). Various metrics were recorded during the SLDJ, including flight time, which we then used to calculate jump height. Additionally, GCT was measured as the duration between the initial landing and the take-off. The reactive strength index (RSI) was also determined, employing the equation FT/GCT (Bishop et al., 2022a). The best result of the three executed jumps on each leg was considered for sbsequent analyses.

### 10-m Linear Sprint

We assessed sprint speed using a 10-m sprint test. Cones clearly marked the beginning and the end of the test. To standardize the setup, the players' front foot was placed 0.5 m in front of the first timing gate. Participants completed two trials separated by a 3-min rest interval. We measured their performance using photoelectric cells (Witty, Microgate, Bolzano, Italy). The timing gates were positioned 1.3 m above the ground and spaced 1.5 m apart (Yeadon et al., 1999). The lower time of the two trials was used for analysis.

### Statistical Analysis

Statistical analyses were conducted using SPSS (Version 25 for Windows; SPSS Inc., Chicago, IL, USA). The mean and standard deviation (SD) were calculated for each variable. The Shapiro- Wilk test was employed to assess the normality of the data, indicating that some inter-limb asymmetry values did not follow a normal distribution. To evaluate the within-session reliability of the test measurements, we used a two-way random intraclass correlation coefficient (ICC) with absolute agreement (95% confidence intervals) and the coefficient of variation (CV). Intraclass correlation coefficients (ICCs) were classified as follows: greater than 0.9 indicated excellent reliability, 0.75–0.9 indicated good, 0.5–0.75 indicated moderate, and below 0.5 indicated poor reliability (Koo and Li, 2016). A CV value was deemed acceptable when it was under 10% (Cormack et al., 2008).

For the purpose of identifying asymmetry between limbs, the asymmetry index (ASI) was calculated using the following formula (Bishop et al., 2016; Impellizzeri et al., 2007):


$$\text{ASI\%= }\frac{\text{Highest}\;\text{Performing}\;\text{Limb}\;\text{-}\;\text{Lowest}\text{​}\text{Performing}}{\text{Highest}\;\text{Performing}\;\text{Limb}}\times 100$$

We defined the highest performing limb (HPL) as the limb with the higher value for each trial and the lowest performing limb (LPL) as the side with the lower value. Paired sample Wilcoxon tests were used to compare the HPL and the LPL. Cohen’s *d* effect size (ES) with 95% confidence intervals was used to quantify the magnitude of the difference between the HPL and the LPL (Cohen, 1988). Values were interpreted as: < 0.20 = trivial, 0.20–0.60 = small, 0.61–1.20 = moderate, 1.21–2.0 = large and > 2.0 = very large, following Hopkins et al. (2009).

An “IF function” was added to the end of the formula in Microsoft Excel to establish the direction of the asymmetry: *IF(left <right,1,−1) (Bishop et al., 2018). Noting that asymmetries may favour either side depending on which limb scores larger, Kappa coefficients (κ) were used to determine agreement with regard to how consistently the direction of asymmetry favoured the same side (Bishop et al., 2021a). Kappa values were interpreted according to the classification proposed by Viera and Garrett (2005). The categorization was as follows: ≤ 0 = poor, 0.01–0.20 = slight, 0.21–0.40 = fair, 0.41–0.60 = moderate, 0.61–0.80 = substantial, and 0.81–0.99 = almost perfect (Cohen, 1960). This interpretation was used because the Kappa coefficient quantifies the level of agreement between two different methods after removing the chance of random agreement (Cohen, 1960).

The 10-m sprint time was subtracted from the COD time in each direction and for each leg to calculate COD deficit time (Freitas et al., 2022; Nimphius et al., 2016):


COD deficit time = COD time - 10m sprint time 

Finally, Spearman’s correlations (*ρ*) were used to compare asymmetry scores on all tests. Statistical significance was established at *p* ≤ 0.05. The magnitude of each correlation was interpreted as follows: trivial (0.00–0.09), small (0.10–0.29), moderate (0.30–0.49), large (0.50–0.69), very large (0.70–0.89), nearly perfect (0.90–0.99), and perfect (1.00) (Hopkins et al., 2009).

## Results

Results of descriptive statistics and reliability measures for the SLCMJ and the SLDJ, the 10-m sprint, COD and COD deficit times are shown in Table 1. Most of the tests demonstrated outstanding within-session reliability, with ICC values of 0.9 or higher. Additionally, tests exhibited acceptable consistency, with CV values below 10%. When comparing HPL and LPL values, significant differences were found in all tasks (*p* < 0.001), with moderate to large effect sizes (ES > 0.6).

**Table 1:** Mean test scores, effect sizes, inter-limb asymmetry values and test reliability data.

Test		Mean ± SD	*p value*	ES	ASI (%)	ICC (95% CI)	CV (%)
COD 70° (s)	HPD	2.27 ± 0.15	< 0.001	1.71	5.09 ± 3	0.94 (0.84–0.98)	6.41
	LPD	2.39 ± 0.15				0.91 (0.78–0.97)	7.52
COD 180° (s)	HPD	2.72 ± 0.15	< 0.001	1.78	3.02 ± 1.73	0.91 (0.74–0.96)	5.41
	LPD	2.80 ± 0.14				0.88 (0.71–0.92)	7.44
COD deficit 70° (s)	HPD	0.33 ± 0.13	< 0.001	1.71	27.8 ± 18.6	0.93 (0.81–0.97)	8.37
	LPD	0.44 ± 0.14				0.90 (0.74–0.96)	9.09
COD deficit 180° (s)	HPD	0.77 ± 0.12	< 0.001	1.78	9.68 ± 5.81	0.84 (0.58–0.94)	11.59
	LPD	0.86 ± 0.11				0.71 (0.23–0.89)	15.33
SLCMJ (m)	HPD	0.18 ± 0.03	< 0.001	1.21	7.33 ± 5.81	0.97 (0.93–0.99)	6.43
	LPD	0.16 ± 0.03				0.95 (0.88–0.98)	5.95
SLDJ (m)	HPD	0.16 ± 0.03	< 0.001	1.07	11.5 ± 12.5	0.96 (0.90–0.98)	9.25
	LPD	0.14 ± 0.04				0.94 (0.87–0.98)	6.5
RSI DJ	HPD	1.36 ± 0.21	< 0.001	1.22	11.16 ± 10.7	0.96 (0.92–0.98)	5.88
	LPD	1.22 ± 0.28				0.95 (0.88–0.98)	6.04
10-m sprint (s)		1.94 ± 0.08				0.88 (0.67–0.95)	4.08

SLCMJ = Single Leg Countermovement Jump; SLDJ = Single Leg Drop Jump; RSI = Reactive Strength Index; COD = Change Direction Capacity; ICC = Intraclass Correlation Coefficient; CI = Confidence Intervals; CV = Coefficient of Variation; ASI = Asymmetry Index; ES = Cohen’s d Effect Size; HPL = Highest Performing Limb; LPL = Lowest Performing Limb

The mean ASI magnitude ranged from 3.02% (COD 180º) to 11.5% (DJ), except for the COD deficit 70º of which the ASI value was 27.8%. Additionally, kappa coefficients and descriptive agreement showed variations in limb performance depending on the test (kappa = −0.26 to 0.44), indicating that asymmetry did not frequently favor the same side among tests.

The only significant relationship in asymmetry scores was between the SLCMJ and COD deficit 70º (*ρ* = 0.54 (0.09–0.81); *p* = 0.03), between the SLDJ and the RSI (*ρ* = 0.80 (0.52–0.93); *p* < 0.01) and between asymmetries in CODs with their subsequent CODs deficit. Figure 4 displays the direction of asymmetry for each test. In this figure, positive values correspond with a right limb advantage in performance, while negative values correspond with a left limb advantage in performance.

**Figure 2:**
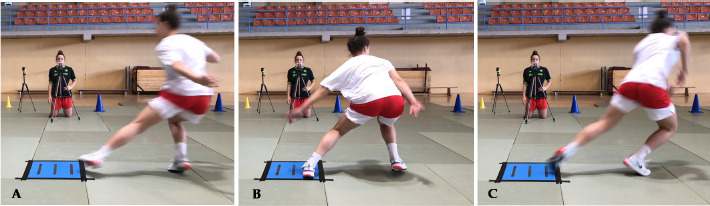
An athlete performing the COD 180° for the left limb.

**Figure 3:**
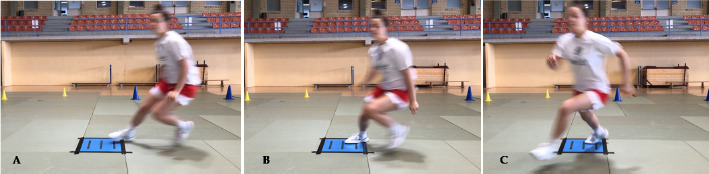
An athlete performing the COD 70° for the right limb.

**Figure 4:**
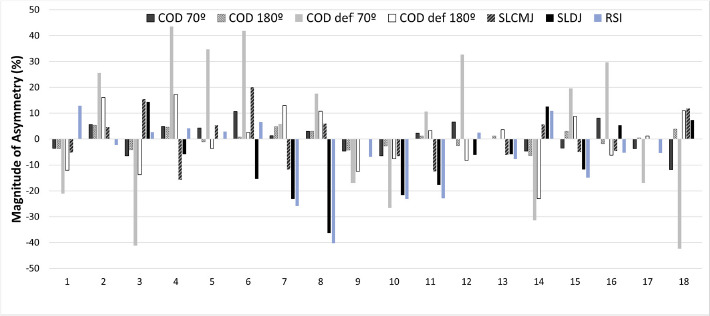
Percentage of asymmetry index (ASI) for each participant and task (positive = right leg dominance; negative = left leg dominance).

Table 2 demonstrates the correlation among the asymmetry magnitude for each task and performance scores of COD variables for the HPL. The results showed significant correlations between time in COD 70º and the ASI from the RSI SLDJ, the SLCMJ, and COD deficit 70º. Time in COD 180º correlated with the ASI from the RSI, the SLDJ, and COD deficit 180º. We found a significant association between asymmetry in COD deficit 70º and GRFy in COD 180º (*ρ* = −0.42 (−0.08–−0.74), *p* = 0.05).

**Table 2:** Spearman’s correlations (*ρ*) between inter-limb asymmetry variables and change of direction performance test scores for the highest performing limb.

Test		ASI COD deficit 70°	ASI COD deficit 180°	ASI SLCMJ	ASI SLDJ	ASI RSI
COD 70°	Time	−0.67**	−0.06	0.51*	0.46*	0.57*
	GRFx (anterior-posterior)	0.03	0.43	0.29	0.09	0.03
	GRFy (mediolateral)	0.09	0.38	0.18	0.22	0.11
	GRFz (vertical)	−0.06	0.41	0.15	0.27	0.28
	GCT	−0.25	−0.18	−0.24	−0.05	0.07
					
COD 180°	Time	−0.20	−0.47*	−0.07	0.57*	0.51*
	GRFx (anterior-posterior)	−0.20	0.29	−0.18	−0.06	−0.25
	GRFy (mediolateral)	−0.42*	−0.33	−0.22	−0.33	−0.36
	GRFz (vertical)	−0.32	0.09	−0.23	−0.11	−0.15
	GCT	0.13	−0.33	0.18	0.23	0.38
					
10-m sprint		0.02	0.07	0.06	0.60**	0.50*

SLCMJ = Single Leg Countermovement Jump; SLDJ = Single Leg Drop Jump; RSI = Reactive Strength Index; COD = Change Direction Capacity; GRF = Ground Reaction Force; GCT = Ground Contact Time; ASI = Asymmetry Index; * (p < 0.05); ** (p < 0.01)

Table 3 shows the correlation between the inter-limb asymmetry magnitude for each task and performance scores of COD variables for the LPL.

**Table 3:** Spearman’s correlations (*ρ*) between inter-limb asymmetry variables and change of direction performance test scores for the lowest performing limb.

Test		ASI COD deficit 70°	ASI COD deficit 180°	ASI SLCMJ	ASI SLDJ	ASI RSI
COD 70°	Time	0.15	0.05	0.48	0.45*	0.47*
	GRFx (anterior-posterior)	0.09	0.49	−0.07	−0.04	−0.06
	GRFy (mediolateral)	0.02	0.29	−0.07	0.04	−0.10
	GRFz (vertical)	0.01	0.36	−0.09	−0.01	−0.03
	GCT	−0.02	0.11	−0.08	0.18	0.13
					
COD 180°	Time	−0.02	−0.07	−0.07	0.64**	0.58*
	GRFx (anterior-posterior)	−0.36	−0.10	−0.15	0.17	0.14
	GRFy (mediolateral)	0.03	−0.01	0.18	−0.60*	−0.57*
	GRFz (vertical)	−0.58*	−0.10	−0.26	0.16	0.23
	GCT	0.14	0.45*	0.03	−0.03	0.23
					
10-m sprint		0.02	0.07	0.06	0.60**	0.50*

SLCMJ = Single Leg Countermovement Jump; SLDJ = Single Leg Drop Jump; RSI = Reactive Strength Index; COD = Change Direction Capacity; GRF = Ground Reaction Force; GCT = Ground Contact Time; ASI = Asymmetry Index; * (p < 0.05); ** (p < 0.01)

The results showed significant correlations between asymmetry in the RSI with time in COD 70º, time in COD 180º and GRFy in COD 180º. The ASI in the SLDJ correlated with time in COD 70º, time in COD 180º and GRFy in COD 180º. We found a significant correlation between asymmetry in COD deficit 180º and GCT in COD 180º (*ρ* = 0.45 (0.03–0.76), *p* = 0.05). Finally, some asymmetry assessments also correlated with linear sprint capacity. The 10-m sprint test correlated with the ASI from the DJ (*ρ* = 0.60 (0.17–0.84), *p* = 0.01) and the ASI from the RSI (*ρ* = 0.50 (0.02–0.79), *p* = 0.04).

## Discussion

We aimed to explore the size and the direction of inter-limb asymmetries in elite youth female basketball players and to investigate the correlation of such asymmetries with COD performance indicators. The main finding of this study was that distinct inter-limb asymmetry scores were correlated with linear and COD performance, partially confirming our hypothesis. The findings further indicated fluctuations in the degree of asymmetry across the tests, with the SLDJ showing the most substantial magnitude of asymmetry.

The first aim of the study was to determine the size and the direction of inter-limb asymmetries in jumping and COD tasks. We found significant differences between which limbs were higher and lower performing in all assessed tasks, ranging from moderate to large effect sizes. Variations in limb performance are frequently observed in athletes participating in team sports, a phenomenon often attributed to the sport-specific demands imposed on the body (Bishop, 2021). It is common for athletes to favour one limb over the other due to its more frequent utilization, leading to enhanced coordination and increased strength in the dominant limb (Stephens et al., 2007). In the context of basketball, the sport's specific requirements include actions such as rebounding jumps, dynamic changes of direction, asymmetrical movements during dribbling, and one-sided passing or throwing actions (Vázquez-Guerrero et al., 2018). The inter-limb asymmetries values ranged from 3.02% (COD 180º) to 11.5% (SLDJ), indicating that there were notable asymmetries in these youth female basketball players, in accordance with other authors reporting inter-limb asymmetries in several athletic populations (Bishop et al., 2018; Fox et al., 2023). It is important to note that, as suggested by previous research, the directionality of asymmetry should also be considered (Bishop et al., 2021a). We found that inter-limb asymmetry values differed across various performance tests, with kappa coefficients ranging from −0.26 to 0.44. This variability in asymmetry percentages depending on the specific task underscores the importance of varying the tasks used to identify and quantify asymmetries as opposed to using a single task. A comprehensive approach that challenges different neuromuscular demands is best for evaluating inter-limb asymmetries in athletes (Arboix-Alió et al., 2021; Bishop et al., 2021a; Madruga-Parera et al., 2020). For example, Figure 2 demonstrates that certain athletes, especially 9 and 10, were consistently left-limb dominant across tasks, which may indicate a need to address deficits in COD and single leg jump performance. Such deficits can be addressed through personalized training programs.

Regarding the second aim of the study, we found significant associations between asymmetry and COD performance, particularly COD times. Asymmetry in the RSI and the SLDJ showed negative correlations with the time taken to complete both COD tests, especially in the 70º COD, for both the HPL and the LPL. This suggests that greater asymmetry in stiffness was associated with slower performance in these COD tasks. Moreover, asymmetry magnitude in the SLCMJ also showed a negative correlation with time in COD 70º for the HPL, reinforcing that asymmetry in lower limb power and strength may negatively influence the ability to efficiently change the direction in similar tasks. Similarly, recent studies have demonstrated that as jump height asymmetries during the SLCMJ increase, acceleration performance decreases (r = 0.49 to 0.59; *p* < 0.05) among female youth soccer players (Bishop et al., 2021b).

Moreover, the correlation analysis also revealed relationships between asymmetry scores and other specific COD performance variables. Indeed, we found significant relationships between asymmetry and GCT for the COD deficit 180º, indicating that players who spent more time in the pivoting action reported a higher asymmetry. Similarly, negative correlations were found between GRFy for the COD 180º and different asymmetry values (SLDJ, RSI, and COD deficit 70º). This finding suggests that athletes who exhibit greater GRFs during pivoting skills tend to display reduced asymmetry in their limb performance. The significant connections found between asymmetry in jumping tasks and COD performance highlight how these physical aspects are linked. The biomechanical and neuromuscular factors causing inter-limb asymmetries in jumping and COD tasks might have shared causes, creating these associations. It is possible that more asymmetry in jumping tasks could change how force is distributed and joints work during COD movements, affecting how efficiently movements are executed (Maloney et al., 2015).

Finally, it is worth noting that some asymmetry scores were correlated with linear sprint ability. For instance, 10-m sprint performance was largely associated with asymmetry values in both the SLDJ (*ρ* = 0.60) and the RSI (*ρ* = 0.50). This suggests that inter-limb asymmetry might have implications not only for COD performance, but also for linear speed, further highlighting the importance of addressing asymmetries in comprehensive training programs (Bishop, 2021). This aspect is especially crucial in youth athletes, such as female basketball players used in the present study, who may experience coordination difficulties due to their tall stature and recent initiation of neuromuscular strength training.

While we find these findings to be of use, our study has some limitations. First, this study focused on a specific population of elite youth female basketball players, and the findings might not be generalized to other age groups or sports. Secondly, we were not able to establish causation due to the study design which was a cross-sectional one. Furthermore, results can vary depending on timing of assessments (Bishop et al., 202b; Fort-Vanmeerhaeghe et al., 2021b). Our assessments were only performed in the post-season. Finally, we did not account for the role of confounding variables, such as injury history, hormonal measures, training history, and pathomechanics.

## Conclusions

We found significant correlations between asymmetry scores and COD times, as well as specific COD performance variables. These results emphasize how asymmetry can negatively impact COD performance, which highlights the importance of correcting asymmetries in training programs for young female basketball players. Coaches, trainers, and practitioners should consider implementing targeted interventions aiming to minimize asymmetry and improve overall limb balance. By doing so, youth elite female basketball players can develop better COD skills and optimize their on-court performance. Additionally, a comprehensive battery of unilateral fitness assessments, encompassing various aspects like jumps, changes of direction, and specific skills, should be considered. Such assessments will provide a holistic view of inter-limb asymmetries, which may allow for the creation of personalized training interventions.
